# Phosphoinositide 3-Kinase Signaling Pathway in Pancreatic Ductal Adenocarcinoma Progression, Pathogenesis, and Therapeutics

**DOI:** 10.3389/fphys.2018.00335

**Published:** 2018-04-04

**Authors:** Divya Murthy, Kuldeep S. Attri, Pankaj K. Singh

**Affiliations:** ^1^Eppley Institute for Research in Cancer and Allied Diseases, University of Nebraska Medical Center, Omaha, NE, United States; ^2^Department of Pathology and Microbiology, University of Nebraska Medical Center, Omaha, NE, United States; ^3^Department of Genetics, Cell Biology and Anatomy, University of Nebraska Medical Center, Omaha, NE, United States; ^4^Department of Biochemistry and Molecular Biology, University of Nebraska Medical Center, Omaha, NE, United States

**Keywords:** PI3K, pancreatic cancer, mucins, MUC1, cancer metabolism, tumor microenvironment, cancer therapy

## Abstract

Pancreatic ductal adenocarcinoma (PDAC) is a highly aggressive malignancy characterized by its sudden manifestation, rapid progression, poor prognosis, and limited therapeutic options. Genetic alterations in key signaling pathways found in early pancreatic lesions are pivotal for the development and progression of pancreatic intraepithelial neoplastic lesions into invasive carcinomas. More than 90% of PDAC tumors harbor driver mutations in *K-Ras* that activate various downstream effector-signaling pathways, including the phosphoinositide-3-kinase (PI3K) pathway. The PI3K pathway also responds to stimuli from various growth factor receptors present on the cancer cell surface that, in turn, modulate downstream signaling cascades. Thus, the inositide signaling acts as a central node in the complex cellular signaling networks to impact cancer cell growth, motility, metabolism, and survival. Also, recent publications highlight the importance of PI3K signaling in stromal cells, whereby PI3K signaling modifies the tumor microenvironment to dictate disease outcome. The high incidence of mutations in the PI3K signaling cascade, accompanied by activation of parallel signaling pathways, makes PI3K a promising candidate for drug therapy. In this review, we describe the role of PI3K signaling in pancreatic cancer development and progression. We also discuss the crosstalk between PI3K and other major cellular signaling cascades, and potential therapeutic opportunities for targeting pancreatic ductal adenocarcinoma.

## Introduction

Pancreatic cancer is the third-leading cause of cancer-related morbidity in the United States, owing largely to the short-term survival rates observed for pancreatic cancer patients despite a low incidence rate (Mazure et al., 1997; Siegel et al., 2016; Attri et al., 2017). Pancreatic adenocarcinoma (PDAC), characterized by a strong desmoplastic stromal formation around the cancerous tissues, accounts for more than 90% of pancreatic cancer cases (Mosdell and Doberneck, 1991). In advanced stages of pancreatic cancer development, the primary tumor reaches the surrounding lymph nodes and then disseminates into the distal metastatic organs like liver, lung, and diaphragm (Pour et al., 1991; Attri et al., 2017). The sudden onset of pancreatic cancer combined with rapid progression of the disease accounts for the lack of robust prognostic markers and early-stage diagnostic markers (Kaur et al., 2012; Le et al., 2016). The short-term survival of the pancreatic cancer patients limits the choice of therapeutic interventions available. Gemcitabine remains the standard care of therapy with success rate of 20–30% when used alone and has a greater efficacy when used in a combination therapy based on a multi-targeting approach. However, cancer tissues evolve rapidly and develop resistance to gemcitabine and other associated therapies (Chand et al., 2016). Specifically, cancer cells reprogram their metabolic machinery to confer gemcitabine resistance, making it extremely difficult to treat (Shukla et al., 2017). The current scenario not only demands a search of novel targets that can be utilized in co-therapeutic regimens but also indicates a need for alternative targets in gemcitabine-resistant and gemcitabine-unresponsive patients.

Pancreatic adenocarcinomas are genetically heterogeneous tumors marked by several genetic mutations found in cancer genomes. The oncogenic mutations can either be driver mutations that cause the onset of disease or passenger mutations that amplify the rate of tumor progression through different stages (Makohon-Moore and Iacobuzio-Donahue, 2016). *K-Ras* is the major driver mutation present in more than 90% of the adenocarcinoma patients (Lennerz and Stenzinger, 2015). The *K-Ras* mutations are found in early lesions and are involved in the progression of cancer to invasive metastatic PDAC (Eser et al., 2014). G12D and G12V are the most common *K-Ras* point mutations found in pancreatic cancer patients (Waddell et al., 2015). The genetically engineered mouse models expressing these oncogenic mutations result in constitutive activation of K-Ras, that regulates downstream signaling pathways involved in proliferation, migration, and metastasis of cancer cells (di Magliano and Logsdon, 2013). The passenger mutations frequently observed in tumor-suppressor genes *CDKN2A, TP53*, or *SMAD4*, and oncogenes *ERBB2* and *EGFR*, accelerate the formation and progression of invasive pancreatic lesions (Waddell et al., 2015; Notta et al., 2016). The other commonly observed mutations in signaling pathway genes, metabolic genes, and other regulatory factors can also act as passenger mutations to aid in the rapid progression of the disease (Jones et al., 2008; Hardie et al., 2017). The convergent target of these genetic alterations is aberrant signaling observed in pancreatic cancer cells. Targeting these aberrant signaling pathways can provide alternative foci and thus help reduce the variability in the effects of pancreatic cancer treatment modalities.

Signaling pathways are critical for maintaining biological functions of all cellular subtypes in both healthy and cancer cells. Deregulation of these interconnected cellular networks results in a myriad of disease conditions, including cancer (Murthy et al., 2013; Creixell et al., 2015). Aberrant cellular signaling also regulates the other molecular hallmarks of cancer such as evasion of growth suppressors, resisting cell death, replicative immortality, angiogenesis, invasion and metastasis, tumor metabolism, and tumor-promoting immune modulation (Hanahan and Weinberg, 2000; Fouad and Aanei, 2017). The mutant K-Ras signaling in pancreatic cancer patients regulates the downstream inositide signaling pathway (Pirhonen et al., 1990; Jones et al., 2008). Phosphoinositide 3-kinase (PI3K) activity is important for cellular proliferation, protein synthesis, apoptosis, migration, metabolism, cytoskeletal rearrangement, response to growth factors, and malignant transformation (Yuan and Cantley, 2008). PI3K controls these functions by regulating a multitude of downstream signaling cascades such as the mechanistic target of rapamycin (mTOR), nuclear factor kB (NF-κB), glycogen synthase kinase 3 beta (GSK3β), p27 and Bad-Bax pathways (Arcaro and Guerreiro, 2007). In addition to K-Ras, the PI3K can be activated by a variety of oncogenic mutations and growth factor receptors present on the surface of cancer cells. While PI3K inhibitors alone have shown limited success in treating pancreatic cancer patients, the use of PI3K inhibitors in combination with other drugs has shown promising results and are currently in clinical trials.

Mutations present in PI3K pathway genes and other-associated regulated pathway genes contribute to tumorigenesis. The PI3KCA gene mutations present in 3–5% of pancreatic cancer patients can act as activating mutations to initiate pancreatic tumor formation and drive its progression and growth (Payne et al., 2015). In addition to their role in cancer cells, PI3K pathway also regulates key cellular functions of immune cells and plays a pivotal role in the interaction of tumor cells with immune cells. Thus, apart from their classical functions, PI3K also regulates metabolic attributes of cancer cell and tumor microenvironment-mediated regulation of tumor growth and survival (Landis and Shaw, 2014; Okkenhaug et al., 2016).

This review summarizes recent advances in the understanding of the regulation of the Class I PI3K signaling pathway (hereafter referred to as PI3K unless otherwise mentioned) in pancreatic cancer, with a particular emphasis on pancreatic cancer-specific regulators and mutations that modify PI3K-mediated functions. The recent literature also highlights an emergent role of the PI3K signaling network in the regulation of tumor metabolism and immune cell function. Further, this review discusses the scope and progress toward developing PI3K-targeted therapies for the treatment of pancreatic cancer.

## Crosstalk between inositide pathway and other key signaling cascades in pancreatic cancer

### The PI3K signaling pathway

**Figure 1:**
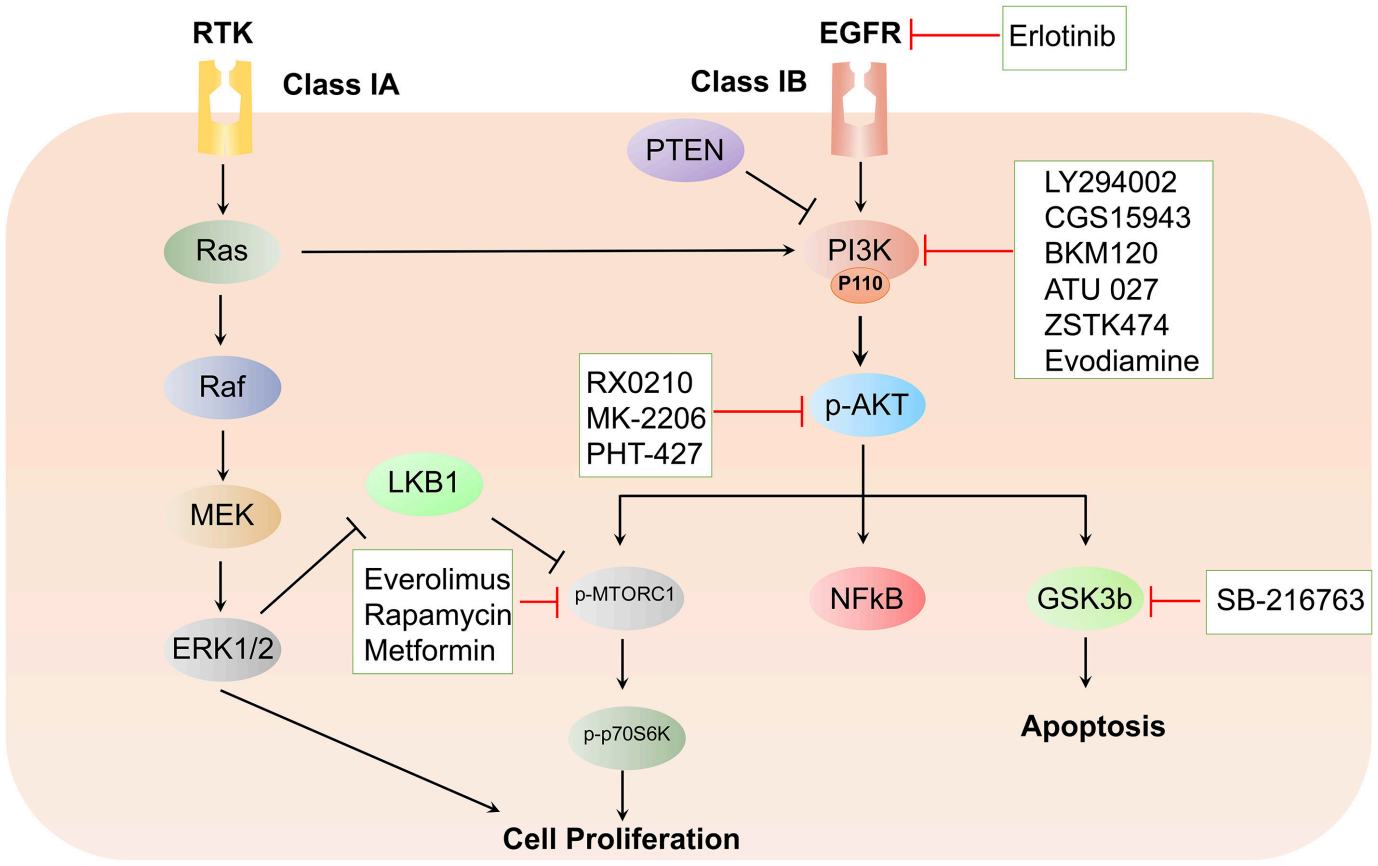
Schematic representation of the PI3K/Akt signaling cascade and targeted therapeutic interventions in pancreatic cancer.

The PI3K/Akt survival pathway is a key downstream target of the family of the rat sarcoma virus (Ras), which are proteins primarily involved in cell proliferation. It is estimated that at least 50% of all cancer patients and 60% of all PDAC patients have deregulation of the PI3K/Akt signaling pathway (Bondar et al., 2002; Schlieman et al., 2003; Yuan and Cantley, 2008; Schild et al., 2009). PI3Ks belong to the lipid kinase family that respond to signals from the Ras family, as well as the receptor tyrosine kinases, and regulate diverse cellular functions including cell transformation, proliferation, growth, motility, and survival (Cantley, 2002; Vanhaesebroeck et al., 2010) (Figure 1). Moreover, the PI3K pathway has been reported to inhibit cellular apoptosis to stimulate cell proliferation in cancer cells (Mao et al., 2016).

The PI3K functions are mediated by diverse phosphorylated forms of phosphatidylinositols (PtdIns) that perform regulatory functions by binding and recruitment of various phosphoinositide binding domain-containing effector proteins. PI3Ks have been classified into three groups that are expressed in most pancreatic cancer cell lines (Edling et al., 2010). Two classes of PI3K class I have been described in this review, namely class IA and class IB. While class IA is composed of three isoforms of the catalytic subunit known as p110α, p110β, and p110δ, class IB encompasses only p110γ. In addition to differences in composition, there are variations in the cues essential for the activation of subclasses IA and IB. While subclass IA (PI3K α, β, and δ) is activated by receptor tyrosine kinases (RTKs), subclass IB (PI3Kγ) is activated by G-protein-coupled receptors (GCPRs). The PI3K signaling cascade is activated by stimulation of RTKs, which results in the recruitment of PI3Ks to the auto-phosphorylated tyrosine residues on RTKs. The interaction between the Src Homology 2 (SH2) domain in the adaptor subunit with PI3K propels the allosteric activation of the catalytic subunit. Activated Class I PI3Ks catalyze the phosphorylation of PtdIns moieties PtdIns(4,5)P_2_ (hereafter referred to as PIP_2_) at their 3′-OH position to generate phosphatidylinositol-3,4,5-triphosphate (hereafter referred to as PIP_3_). The resultant second messenger PIP_3_ further recruits pleckstrin homology-(PH) domain-containing signaling proteins to the cancer cell membrane. One such protein is the serine/threonine kinase, phosphoinositide-dependent kinase 1 (PDK1), which is a known activator of Akt/protein kinase B (PKB) (Falasca and Maffucci, 2007; Lien et al., 2017).

The Class II PI3K (PI3KC2) subfamily comprises of three members (in vertebrates), namely, PI3KC2 α, β, and γ (Jean and Kiger, 2014). While the N terminal domain of PI3KC2 has been demonstrated to interact with adapter proteins that control distinct steps of vesicular trafficking processes such as endocytosis and vesicle recycling, the C terminal region binds to PIP_2_-containing membranes allowing membrane recruitment of PI3KC2α (Stahelin et al., 2006; Posor et al., 2013). The Class III PI3K, Vps34, catalyzes the synthesis of PtdIns(3)P at distinct intracellular membranes and plays a critical role in regulation of endosomal protein sorting, autophagosome formation, endosome-lysosome maturation, and cytokinesis (Sagona et al., 2010; Jaber et al., 2012; Ma et al., 2014). Among the different classes of the PI3K family, the Class I PI3Ks have been widely studied and implicated in pancreatic cancer, and henceforth, will be the focus of this review.

### Akt signaling

The serine-threonine kinase Akt belongs to the AGC kinase family and is the central signaling molecule that regulates diverse cellular processes critical for cell survival and cell cycle progression (Yamamoto et al., 2004). The Akt kinase family includes Akt1, Akt2, and Akt3, which display distinct features despite extensive structural homology. Interestingly, Akt was originally identified as a regulator of insulin receptor signaling. Akt is composed of the following three domains: (1) an N-terminal pleckstrin homology PH domain, (2) a C-terminal extension (EXT) domain containing a regulatory hydrophobic motif (HM), and (3) a central kinase catalytic subunit (CAT) domain (Kumar and Madison, 2005). Upon PI3K activation, Akt is recruited to the plasma membrane through the interaction of the pleckstrin homology domain with membrane lipids (James et al., 1996). The recruited Akt is phosphorylated by PDK1 at residues Thr308 and by mTORC2 at Ser473 present in the activation loop (Alessi et al., 1997; Sarbassov et al., 2005). The initiation of the Akt signaling cascade stimulates NF-kB, hypoxia-inducible factor-1α (HIF-1α), and Forkhead transcription factors (FoXO), in addition to regulation of the cell cycle and inhibition of apoptosis (Cardone et al., 1998; Ayabe, 1999; Brunet et al., 1999; Zhong et al., 2000). The Akt isoforms carry specific genetic aberrations in diverse types of tumors, which positively correlate to cancer aggressiveness and poor prognosis. Such genetic alterations underline the discrete functional role of Akt in pancreatic cancer development and progression.

### mTOR signaling

The kinase mTOR, another serine/threonine kinase, functions downstream of PI3K signaling. Furthermore, mTOR regulates a wide array of growth-related functions by increasing cell proliferation and survival, protein degradation, and reorganization of the actin cytoskeleton (Foster and Fingar, 2010). While enhanced phosphorylation of ribosomal protein S6 kinase beta-1 (P70S6K) aids in protein synthesis, binding to eukaryotic translation initiation factor 4E (eIF4E) is abolished by phosphorylation of the eIF4E-binding proteins (4E-BPs) to relieve translational block, which leads to protein translation and cell growth (Gingras et al., 1998). Two distinct complexes of mTOR exist within the cells. While mTORC1, which is composed of mTOR, Gbl, and Raptor, transcends signals following PI3K-Akt activation, mTORC2, which is composed of mTOR, Gbl, and Rictor, is involved in full activation of Akt, thus promoting cell proliferation (Hay and Sonenberg, 2004). The activation of mTOR regulates translation of different proteins, including cyclin D1, which regulates cell cycle progression, and HIF-1α, which regulates expression of the pro-angiogenic vascular endothelial growth factor (VEGF) (Grewe et al., 1999). Because mTORC1 is highly sensitive to rapamycin, this family of molecules epitomizes the first-generation of mTOR inhibitors. Efforts are focused on inhibiting the mTORC1 complex, and little attention has been paid to mTORC2, which is largely insensitive to rapamycin. However, in a recent study by Driscoll et al. deletion of the obligate mTORC2 subunit Rictor delayed tumorigenesis. The study further demonstrated the utilization of combined inhibition of mTORC1/2 and PI3K as a potential therapeutic strategy to inhibit the progression of pancreatic cancer (Driscoll et al., 2016).

### PI3K regulation mediated by PTEN

Being one of the most frequently disrupted tumor suppressors in cancer, a mutation in the phosphatase and tensin homolog deleted on chromosome 10 (PTEN), the natural antagonist of PI3K, relieves the repression of the PI3K/Akt signaling axis in PDAC. Investigators have observed that the development of pre-malignant PanIN in Pdx1-Cre; K-Ras^G12D/+^ mice with conditional deletion of *Pten* was accelerated and accentuated the phenotype of acinar-to-ductal metaplasia (ADM) (Stanger et al., 2005; Hill et al., 2010). In principle, the PTEN phosphatase dephosphorylates PIP_3_ to PIP_2_ and reduces tumor cell growth and survival (Maehama and Dixon, 1998; Cantley and Neel, 1999; Di Cristofano and Pandolfi, 2000; Asano et al., 2004). Additional studies have shown that loss of PTEN expression in 25–70% of cases is concurrent with the short-term overall survival (Asano et al., 2004; Ying et al., 2011). Activation of the NF-κB pathway and its downstream cytokine network had been identified as a key altered pathway on combined oncogenic deletion of *K-Ras* and *Pten*. Aberrations in the PTEN/PI3K pathway are thus frequently observed in PDAC which results in activation of tumor-promoting stromal and immune cell populations that shape the PDAC tumor microenvironment (Ying et al., 2011). VEGF, predominantly known to promote tumor angiogenesis, is also inversely regulated by PTEN in pancreatic cancer cells (Ma et al., 2009).

## Role of K-Ras signaling in the progression of pancreatic cancer

K-Ras belongs to the Ras family of guanosine-5′-triphosphatases (GTPases). Activating *K-Ras* mutations, mainly in codon 12, are the first genetic changes detected during the progression of pancreatic cancer and are present in 75–90% of all pancreatic adenocarcinomas (Shibata et al., 1990; Dergham et al., 1997; Wang et al., 2002). Oncogenic K-Ras activates a plethora of signaling pathways associated with the survival of cancer cells. Such a characteristic suggests that K-Ras signaling is an ideal drug target to counteract the progression of pancreatic cancer. Classically, growth factor-mediated exogenous stimulation results in activation of Ras GTPases, which dimerize and further regulate downstream effector molecules. Attempts to identify critical Ras effectors in pancreatic duct epithelial cell systems have revealed a dependency of K-Ras on the PI3K/Akt signaling cascade. It is well-established that the PI3K/Akt pathway is activated in human PDAC as well as K-Ras-driven mouse models of pancreatic cancer (Jimeno et al., 2008; Kennedy et al., 2011; Eser et al., 2013). The various mouse models utilized for understanding the role of PI3K have been discussed in Table 1. A recent study, which utilized an *in vivo* genetic model, demonstrated a critical role of the K-Ras-PI3K-PDK1 axis in mediating ADM, PDAC formation, and maintenance. The enhanced ducts formed from the acinar cells further develop PanIN lesions (Baer et al., 2014). Activation of K-Ras by interaction with the protein-coding gene heterogeneous nuclear ribonucleoprotein A2/B1 (HNRNPA2B1) is associated with upregulation of the mTOR signaling pathway and results in PDAC cell survival and tumor formation in mice (Barcelo et al., 2014). Other than directly activating the PI3K signaling cascade, increased interaction between the K-Ras 4B isoform with calmodulin via the hypervariable region indirectly modulates PI3K signaling (Nussinov et al., 2015). Reactive oxygen species (ROS) is an important determinant of pancreatic cancer pathogenesis. Oncogenic K-Ras-driven metabolic and signaling alterations regulate the production of ROS in pancreatic cancer (Wang et al., 2015; Storz, 2017). Furthermore, the membrane translocation and activation of ROS-producing family of enzymes, namely NADPH oxidases (NOX), is mediated by the PI3K signaling. NOX activation mediates the pro-survival effects of ROS by sustained phosphorylation of JAK2 and by suppressing apoptosis (Lee et al., 2007). Akt plays a direct role in the activation of NOX proteins through NFkB-mediated upregulation of the NOX subunit p22^*phox*^ (Edderkaoui et al., 2013).

**Table 1:** Mouse models of pancreatic cancer utilized to understand the role of phosphoinositide signaling pathway in pancreatic cancer.

**Genotype**	**Mutation in PI3K gene**	**Effect of the mutation in pancreatic cancer**	**References**
Pdx-1-Cre; LSL-Kras^G12D^	None	ADM and PanIN lesions observed in all animals	Baer et al., 2014
Pdx-1-Cre; LSL-Kras^G12D^, p110α^+/lox^	Genetic inactivation of one allele of the kinase domain of pancreatic p110α	ADM and PanIN lesions observed in most animals	Baer et al., 2014
Pdx-1-Cre; LSL-Kras^G12D^, p110α^lox/lox^	Genetic inactivation of the kinase domain of pancreatic p110α	All types of pancreatic lesions blocked	Baer et al., 2014
Pdx2-Cre; LSL-Kras^G12D^; LSL-Trp53^R172H/+^	None	Developed primary PDAC	Eser et al., 2013
Pdx1-Cre; LSL-Kras^G12D^; p53^lox/+^,	None	Developed primary PDAC	Baer et al., 2014
Pdx1-Cre; LSL-Kras^G12D^; p53^Lox/+^,p110α^+/lox^	Genetic inactivation of one allele of the kinase domain of pancreatic p110α	Blocked acinar to ductal plasticity. Low-grade PanIN lesions formed, Stalled the progression of low-grade PanINs toward high-grade PanINs	Baer et al., 2014
Pdx1-Cre; LSL-Kras^G12D^; p53^lox/+^, p110α^lox/lox^	Genetic inactivation of the kinase domain of pancreatic p110α	Blocked acinar to ductal plasticity. Low-grade PanIN lesions observed, Stalled the progression of low-grade PanINs toward high-grade PanINs and adenocarcinoma	Baer et al., 2014
Ptf1a^Cre/+;^ LSL-Kras^G12D/+^	None	Acini-derived tumors did not undergo ADM	Baer et al., 2014
Ptf1a^Cre/+;^ LSL-PIK3CA^H1047R/+^	Activating mutation (H1047R) in the catalytic domain of PIK3CA	Pancreatic size and weight increased, induced ADM and premalignant PanINs	Eser et al., 2013
Pdx1-Cre, LSL-Kras^G12D^; PTEN^lox/lox^	Disruption of PTEN gene	Accelerated pre-malignant PanINs and PDAC; No expression of senescent markers; Accentuated ADM	Hill et al., 2010; Kennedy et al., 2011
Pdx1-Cre; Pten^lox/lox^	Deletion of PTEN gene	Increased proliferation and centro-acinar cell expansion	Stanger et al., 2005

It is interesting to note that pharmacological inhibition of the protein kinase cascade pathway known as the mitogen-activated protein kinase/extracellular signal-regulated kinases (MAPK/ERK), one of the other well-established mediators of K-Ras-dependent cancer progression, remains ineffective in reducing the tumor burden in K-Ras-mutant cancers (Hayes et al., 2016). However, this recent study by Hayes et al. revealed dynamic reprogramming of signaling networks that resulted in ERK activation. Thus, ERK inhibition is a potential therapeutic approach in K-Ras-dependent pancreatic cancer. Further, the study by Hayes et al. also showed the regulatory roles of PI3K/Akt/mTOR signaling in the sensitivity of the ERK inhibitor. Therefore, co-targeting the ERK signaling pathway along with the PI3K signaling may bestow a distinct and advantageous therapeutic strategy for pancreatic cancer.

## Regulatory interaction between mucins and phosphoinositide signaling

A characteristic feature that defines pancreatic cancer is the aberrant overexpression of a high molecular weight glycoproteins, mucins (MUC). Mucins, such as MUC4, have been demonstrated to be the targets of K-Ras^G12D^ mutant, which is regulated by a transcriptional or post-transcriptional mechanisms (Vasseur et al., 2015). Furthermore, PI3K signaling is regulated by MUC1, a type I transmembrane glycoprotein that regulates aggressiveness in PDAC by inducing metabolic and signaling alterations (Chaika et al., 2012a; Liu et al., 2014; Mehla and Singh, 2014; Gebregiworgis et al., 2017; King et al., 2017). MUC1 regulates the expression and signaling of multiple receptor tyrosine kinases, including PDGFR, EGFR, and c-Met that signals through the PI3K signaling cascade to drive cellular processes such as proliferation, migration, and survival (Singh and Hollingsworth, 2006; Singh et al., 2007, 2008; Engel et al., 2016). Also, MUC1 mediates the nuclear localization of EGFR to influence the interaction between EGFR and transcriptionally active promoter regions (Bitler et al., 2010). Interestingly, an intimate link between MUC1 cytoplasmic tail expression and the activation of the PI3K-Akt pathway has been observed previously in fibroblasts and thyroid cancer cells (Raina et al., 2004). In accordance with these previous findings, a recent study on non-small cell lung cancer also showed that the interaction between the cytoplasmic domain of MUC1 with the SH2 domain of PI3K p85 subunit is critical for the activation of the PI3K-Akt-mTOR pathway (Kato et al., 2007). Additionally, multidrug resistance is a prominent phenomenon in pancreatic cancer that is modulated by the upregulation of transporters of the ATP-binding cassette (Nath et al., 2013). MUC1 has been further shown to regulate the expression of the multidrug-resistance genes by Akt-dependent and -independent pathways conferring the multidrug-resistance phenotype in pancreatic cancer cells (Nath et al., 2013). It is also pertinent that MUC1-mediated resistance to radiotherapy and chemotherapy may be governed by PI3K signaling (Gunda et al., 2017; Shukla et al., 2017). Therefore, targeting the regulatory axis of MUC-PI3K signaling could be a promising therapeutic strategy for pancreatic cancer.

## Genetic alterations in PI3K signaling pathway in PDAC progression

PI3K and its downstream effectors are constitutively activated in K-Ras-driven pancreatic cancer. p110α, the PI3K class IA subunit, is encoded by *PIK3CA* and encompasses hotspot mutations in the helical (E542K and E545K) and catalytic domains (H1047R). Such oncogenic mutations result in constitutive activation of the PI3K signaling, as reported in breast and lung cancers (Bader et al., 2005; Liu et al., 2009). Despite the sparse occurrence of activating mutations in p110α in PDAC, the enhanced expression of activated p110α mimics mutated K-Ras-mediated oncogenesis (Schonleben et al., 2006; Jones et al., 2008; Biankin et al., 2012; Eser et al., 2013). When expressed specifically in the pancreas, p110α^H1047R^ induces PI3K activation, leading to enhanced ADM and PanIN formation. Overexpression of p110α^H1047R^ phenocopies mutant K-Ras driven PDAC and is independent of cross-activation of K-Ras (Engelman et al., 2008; Adams et al., 2011; Liu et al., 2011). A subsequent study showed the activation of Akt and GSK3 in a KC mice model (Eser et al., 2013). Along these lines, pancreas-specific expression of kinase-dead p110α isoform inhibited initiation of pancreatic pre-neoplastic lesions. In contrast, the PI3K activity of pancreatic p110β is dispensable for oncogenic K-Ras-induced cancer formation (Baer et al., 2014). In opposition to these findings, Collisson et al. reported the inability of p110α^H1047R^ to induce PanIN and PDAC formation using a Pdx1-CreER mouse line, an alternate murine model utilized to study pancreatic cancer formation and progression. In addition to studies on p110α, a report by Edling et al. highlighted the role of p110γ in oncogenic transformation of pancreatic cells. This study shows increased expression of p110γ in PDAC tissue compared with normal ducts. Further, depletion of p110γ resulted in reduced cell proliferation, emphasizing the participation of p110γ in pancreatic cancer progression (Edling et al., 2010).

Constitutive activation of PI3K-effector Akt is an indicator of the aggressiveness of pancreatic cancer (Edling et al., 2010; Massihnia et al., 2017). In addition to activated Akt in general, *Akt2* amplification has been observed in 10–32% of pancreatic adenocarcinomas and contributes to the malignant phenotype in a subset of human PDAC patients (Cheng et al., 1996; Altomare and Testa, 2005). Moreover, amplification of *Akt2* has been identified in studies that apply comparative genomic hybridization (array CGH; Liang et al., 2014). Likewise, Akt2 has long been implicated in signaling pathways downstream of various mitogenic growth factors critical in the development of pancreatic cancer (Ruggeri et al., 1998). Interestingly, PDAC hetero-transplants that possess mutant *K-Ras* and *Akt2* amplification are extremely responsive to co-treatment with dactolisib (BEZ235) and panobinostat, resulting in the inhibition of tumor growth (Venkannagari et al., 2012). Mutations and amplification of *Akt* are thus instrumental in determining the oncogenic landscape of pancreatic cancer.

In addition to amplified *Akt*, also observed in pancreatic tumor cell lines is decreased *PTEN* expression accompanied by an elevation in PI3K/Akt signaling. The poor expression of *PTEN* is shown to be a result of promoter methylation (Asano et al., 2004). Previous studies show the significantly low frequency of deletion or loss-of-function mutations targeting *PTEN* in human PDAC (Asano et al., 2004). However, recent human PDAC genome analyses, along with mouse genetic studies, have revealed frequent deletion of the *PTEN* gene in pancreatic tumor specimens, leading to the activation of NF-κB and its downstream cytokine pathway, which is associated with shaping the tumor microenvironment in PDAC (Ying et al., 2011). Further, a multiplatform-based survey was performed to study alterations in the PI3K/Akt/mTOR pathways in over 1,000 cancer cases. Analyses revealed that around 1% of the patients harbored non-silent somatic mutations in the components of the PI3K/Akt/mTOR pathways, < 1% encompassed copy number loss, and 2–5% contained amplification of Akt2, which is consistent with a previous finding (Zhang et al., 2017). Taken together, it is evident that the PI3K/Akt/PTEN signaling loop is a critical signaling hub, which is altered during PDAC initiation and progression.

## Inositide signaling pathway-mediated metabolic regulation

Pancreatic cancer is characterized by altered metabolic pathways rewired predominantly by the mutant K-Ras. One of the key metabolic changes driven by oncogenic K-Ras involves elevated glucose uptake (Ying et al., 2012; Kerr et al., 2016). In addition to enhanced glycolysis, mutant K-Ras is responsible for the shuttling of glycolytic intermediates to various anabolic pathways such as pentose phosphate pathway and hexosamine biosynthetic pathway, which are critical for the genesis, proliferation, and progression of pancreatic cancer (Ying et al., 2012). The reprogramming of glutamine metabolism and the dependence of PDAC cells on this non-canonical pathway for supporting pancreatic cancer growth is also contingent on oncogenic K-Ras (Son et al., 2013). In addition to its role in regulating anabolic glucose metabolism, K-Ras drives transcriptional reprogramming to elevate the expression of autophagic and macropinocytosis-associated genes in order to meet the metabolic requirements of the cell (Yang et al., 2011; Commisso et al., 2013).

The engagement of K-Ras with various metabolic pathways is primarily mediated by PI3K/Akt and MAPK (Deprez et al., 1997; Barthel et al., 1999). Such regulatory alterations of key metabolic factors are strongly associated with the Akt signaling pathway. Oncogenic K-Ras can enhance the activity of the metabolic enzyme ATP citrate lyase (ACLY) in an Akt-dependent manner, resulting in increased histone acetylation (Lee et al., 2014). Altered histone acetylation can impinge upon cellular metabolism by altering the cellular acetyl-CoA pool, expression patterns of genes, response to DNA damage in cancer cells, and DNA replication (Unnikrishnan et al., 2010; Sulli et al., 2012). The role of PI3K/Akt has been well established in lung adenocarcinoma and hepatoma cells wherein PI3K-mediated enhanced expression levels and membrane localization of GLUT1 were observed (Barthel et al., 1999; Makinoshima et al., 2015). In the case of pancreatic cancer, treatment with the PI3K inhibitors LY294002 and wortmannin led to a significant decrease in the expression of GLUT1 and subsequently, a reduction in the glucose uptake by cells (Melstrom et al., 2008). While stimulation of phosphofructokinase enzymatic activity by Akt has been observed, Akt mediates regulation of the mitochondrial localization of the glycolytic enzymes Hexokinase (HK)1 and HK2 in Rat1a fibroblasts (Majewski et al., 2004).

The PI3K/Akt signaling pathway also regulates the HIF-1α, one of the master regulators of PDAC metabolism (Chaika et al., 2012a; Kang et al., 2014). Notably, it has been shown that inhibition of PI3K/Akt pathway results in a significant decrease in the expression and the DNA-binding ability of HIF-1α (Kilic-Eren et al., 2013). The strong suppressive effects of the downregulation of K-Ras and downstream signaling in the glycolytic pathway via HIF-1α is evident in diverse tumor cells (Mazure et al., 1997; Fukuda et al., 2002; Gao et al., 2002).

MUC16, known to facilitate PDAC progression, regulates the activity of mTOR and consequently, its downstream target *c-myc*, which is crucial for PDAC growth and metabolism. Reduction in mTOR activity and *c-myc* expression in MUC16-knockdown cells results in global metabolic alterations that significantly reduce the cellular glycolytic and nucleotide metabolite pools (Shukla et al., 2015). Apart from metabolic regulation of *c-myc*, PI3K signaling also controls c-myc protein abundance in a GSK3-dependent fashion in PDAC cells (Schild et al., 2009). Although available studies do not adequately demonstrate the direct role of PI3K signaling in commencing metabolic reprogramming of PDAC cells, future studies directed toward elucidating the interplay between this tumorigenic metabolic pathway with PDAC metabolism will potentially identify new test models and therapeutic options for pancreatic cancer.

## Role of PI3K signaling in the pancreatic tumor microenvironment

PDAC is a very unusual tumor where in the stromal cells outnumber the tumor cells (Erkan et al., 2008). The thick desmoplasia makes the tumor microenvironment hypoxic, acidic, and impermeable to drugs, thus creating a barrier to treatment options (Pandol et al., 2009). Studies performed in this arena have shown that manipulating the tumor microenvironment impacts tumor growth kinetics (Whatcott et al., 2015; Abrego et al., 2017). The pancreatic tumor microenvironment is a heterogeneous compartment, majorly consisting of cancer-associated fibroblasts, different immune cells, stellate cells, endothelial cells, and the extracellular matrix. The dynamicity of the tumor microenvironment is mediated through various signaling factors secreted during tumor and accessory cell crosstalk (Feig et al., 2012). Such a heterocellular process of oncogenic cross-signaling results in increased pancreatic cancer proliferation, metastasis, and altered apoptosis. For instance, the interaction between stromal cells and pancreatic cancer cells has been shown to alter intracellular signaling and metabolic pathways in pancreatic cancer cells (Bailey et al., 2008; Hwang et al., 2008; Behrens et al., 2010; Chaika et al., 2012b; Tape et al., 2016; Rucki et al., 2017). Interestingly, PI3K signaling is also regulated by the cross-exchange of such signaling stimuli (Yuan and Cantley, 2008). While the role of PI3K signaling is well-known in the development and function of different immune cells, the significance of PI3K signaling in the pancreatic cancer tumor microenvironment is currently being examined.

### PI3K signaling in cancer-associated fibroblasts

**Figure 2:**
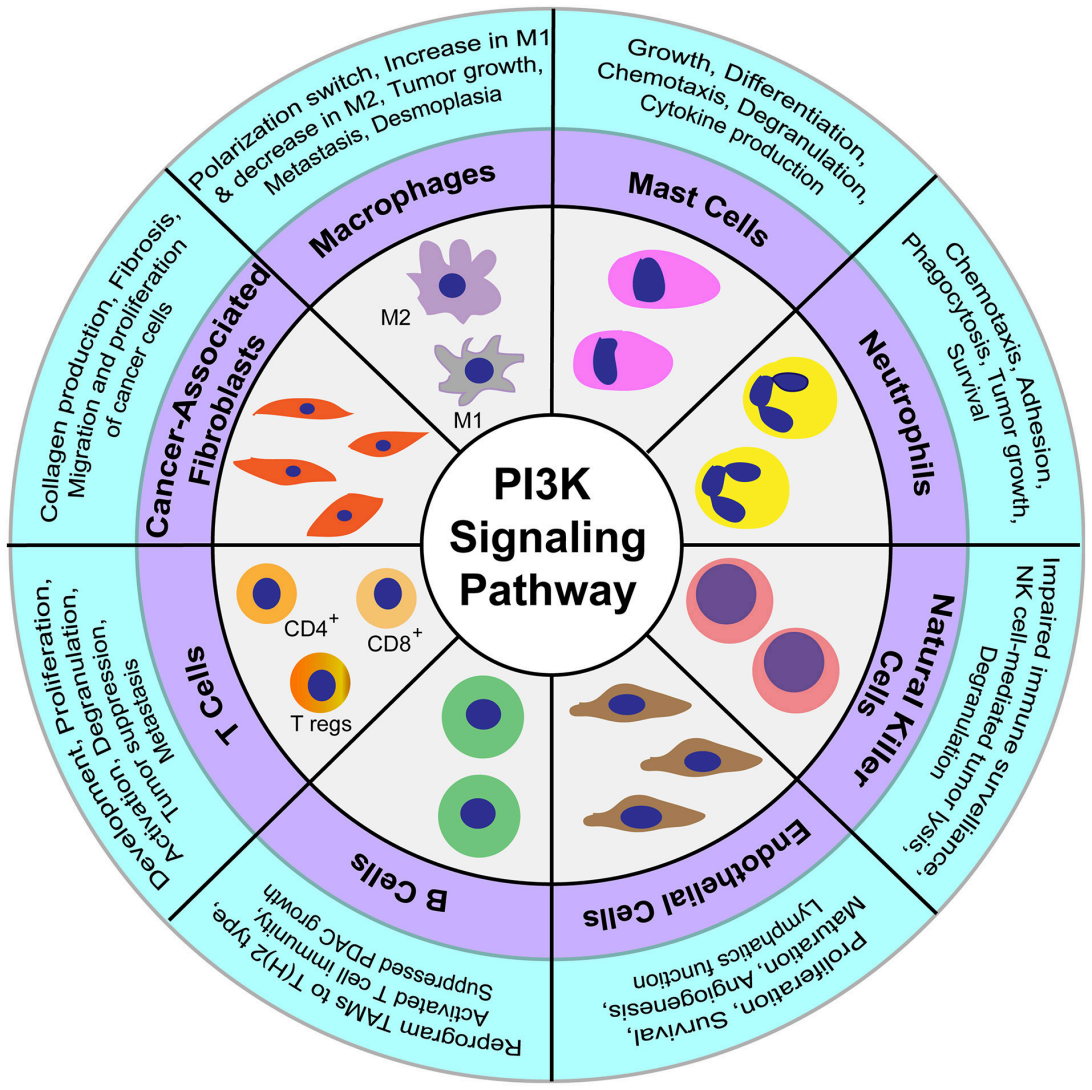
Regulatory functions of PI3K signaling in diverse cellular components constituting the tumor microenvironment.

Cancer-associated fibroblasts are specialized fibroblasts that constitute the majority of the cells present in the tumor microenvironment (Apte et al., 2013). These fibroblasts are mostly derived from stellate cells in pancreatic cancer (Ohlund et al., 2017) and play a secretory role in the tumor microenvironment by releasing a variety of factors like collagen, proteoglycans, glycoproteins, and other components that comprise the extracellular matrix (Shan et al., 2017). Cancer-associated fibroblasts have been shown to protect cancer cells from chemotherapeutic agents, and increase their proliferation and migration *in vitro* and *in vivo* (Xing et al., 2010; Shiga et al., 2015). Similarly, the platelet-derived growth factor secreted by immune cells regulate migration and proliferation of cancer cells by the activation of PI3K signaling pathway (Figure 2; Cho et al., 2016). Another mitogen, cholecystokinin, binds to the receptors present on stellate cells to activate PI3K pathway for regulation of collagen production and fibrosis (Berna et al., 2010; Smith et al., 2014). Pancreatic cancer cells and cancer-associated fibroblasts show a reciprocal release of mitogens from both the cell types that regulate activation of PI3K signaling. This positive loop for PI3K activation is critical for pancreatic cancer progression (Figure 2; Bussard et al., 2016).

### PI3K signaling in innate immune cells

#### Macrophages

Macrophages are phagocytic immune cells involved primarily in the defense against pathogens and in the wound-healing process (Ginhoux et al., 2016). Macrophages can exist as either classically activated—M1 macrophages involved in defense against pathogens or alternatively activated—M2 macrophages involved in wound healing and tissue repair (Yoshikawa et al., 2012; Barros et al., 2013). These two polarized subtypes of macrophages play an antagonistic function in the regulation of pancreatic tumor cell growth. While M1 macrophages are pro-tumorigenic, M2 macrophages are anti-tumorigenic (Karnevi et al., 2014). Notably, the survival, adhesion, metabolism, polarization, and motility of macrophages have been shown to be regulated by PI3K signaling (Luyendyk et al., 2008; Xie et al., 2014; Covarrubias et al., 2016). The PI3K signaling pathway also activates Akt1 and Akt2 kinases, thereby regulating the macrophage polarization switch. Furthermore, Akt1-deficient macrophages were shown to produce M1 macrophages that secrete pro-inflammatory cytokines, and Akt2-deficient macrophages were shown to produce M2 polarized macrophages that express Arg1, Fizz1, and interleukin-10 (IL-10) (Chaudhuri, 2014). Another marker associated with PDAC tumor-associated macrophages is PI3Kγ, a class I PI3K lipid kinase isoform specifically expressed in myeloid cells. Inhibition of PI3Kγ in PDAC-associated macrophages suppresses the expression of M2 macrophage markers, while increasing expression of M1 macrophage markers, thereby leading to the suppression of CD8^+^ T-cell-mediated immunity. Additionally, it has been shown that inhibition of PI3Kγ can suppress tumor growth, invasion, metastasis, and desmoplasia in pancreatic cancer (Figure 2; Kaneda et al., 2016a). In another study, therapeutic targeting of PI3Kγ along with checkpoint inhibitor treatment resulted in tumor suppression and increased survival (Kaneda et al., 2016b). Macrophages also interact with pancreatic stellate cells to regulate fibrosis. Lipopolysaccharide-activated macrophages regulate stellate cell activity through the signaling of transforming growth factor beta (TGF-β) to induce secretion of Th2 cytokines, which in turn polarizes macrophages to the M2 subtype (Schmid-Kotsas et al., 1999; Xue et al., 2015). Moreover, the crosstalk between M1 macrophages and stellate cells, through cytokine signaling, regulates the suppression of pancreatic tumors (Shi C. et al., 2014).

#### Neutrophils

Neutrophils are primary cells recruited to the site of inflammation caused by bacterial infections. These cells migrate to the inflammation sites by sensing chemotactic signals, where the neutrophils release cytokines, chemokines, and proteinases that further recruit macrophages and dendritic cells (Kolaczkowska and Kubes, 2013). Pancreatic cancer cells attract neutrophils into the vicinity of tumor cells and the surrounding stromal compartment (Reid et al., 2011; Chao et al., 2016). The neutrophil influx in tumors correlates with tumor growth and poor prognosis in PDAC patients (Reid et al., 2011; Ino et al., 2013). Moreover, tumor-associated neutrophils promote cancer progression by reactive oxygen species-mediated DNA damage and modulate migration of tumor and other immune cells by secretion of various pro-inflammatory cytokines and chemokines (Scapini et al., 2000; Mangerich et al., 2012). The PI3K signaling pathway in neutrophils is known to regulate survival, growth, adhesion, phagocytosis, and chemotaxis (Figure 2; Engelman et al., 2006; Pinho et al., 2007). However, the exact mechanism and significance of PI3K signaling with respect to neutrophils in PDAC remains elusive.

#### Mast cells

Mast cells primarily associated with an inflammatory allergic reaction, are also found in the pancreatic cancer tumor microenvironment (Ma et al., 2013). Comparison of human pancreatic cancer samples with the normal pancreas showed an elevated number of mast cells, which were associated with reduced patient survival (Strouch et al., 2010). In a study conducted on spontaneous PDAC K-Ras (G12V) mice, the deficiency of mast cells was shown to suppress pancreatic tumor cell growth (Chang et al., 2011). Importantly, the PI3K pathway is critical in regulating growth, differentiation, chemotaxis, degranulation, and cytokine production in mast cells (Figure 2; Kim et al., 2008).

#### Natural killer cells

Natural killer (NK) cells are effector lymphocytic cells of the innate immune system that are known to migrate early to the sites of inflammation during infection. The role of NK cells in cancer is recently being realized, in addition to their classical role in host defense during viral infection (Cerwenka and Lanier, 2016). The cytotoxic activity of NK cells is not only regulated by the extent of receptors present on the cell surface, but also by sensing the availability of ligands in the modified microenvironment (Long et al., 2013). NK cells can elicit their effector function without being educated on the epitopes present in the altered cells or undergoing any clonal selection (Cerwenka and Lanier, 2016). Activating killer receptors NKp30 and NKG2D are two important receptors present on the NK cell surface that are primarily involved in tumor cell recognition and subsequent killing (Peng et al., 2013). Cancer-associated fibroblasts, macrophages, T cells, and other accessory cells present in the tumor microenvironment are also known to modulate expression of the NKG2D receptor and production of interferon gamma (IFNγ) in NK cells (Vitale et al., 2014). Additionally, inhibition of the PI3K signaling pathway in NK cells impairs their function in immune surveillance by preventing their degranulation activity (Jiang et al., 2000). The activity of NK cells is reduced in pancreatic cancers, and the decreased expression of NKG2D, NKp30, and NKp46 correlates with tumor progression in pancreatic cancer patients (Figure 2; Peng et al., 2013, 2014). Furthermore, expression of the receptors CD226 and CD96 in NK cells is associated with cell dysfunction-mediated immune escape and the progression of pancreatic cancer (Peng et al., 2016). In contrast to the tumoricidal function of the receptor NKG2D in NK cells, its expression in cancer cells regulates proliferation and metastasis by activation of PI3K signaling (Benitez et al., 2011; El-Gazzar et al., 2014). Moreover, treatment of pancreatic cancer cells by valproic acid, a histone deacetylase inhibitor, has been shown to promote NK cell-mediated lysis of tumors by activation of PI3K/Akt signaling pathway (Figure 2; Shi P. et al., 2014). Taken together, these studies suggest that PI3K signaling pathway in NK cells is also critical for regulating the pancreatic tumor biology.

### PI3K signaling in adaptive immune cells

#### T cells

Adaptive immune cells are responsible for a range of functions, from long-term memory and pathogenic infections to complex diseases like cancer (Grivennikov et al., 2010). Adaptive immune responses are mostly suppressed in pancreatic cancer due to the evolution of immune escape mechanisms, immunoediting, and the development of mechanisms of immune resistance (Gajewski et al., 2013). CD3^+^ T cells are the key adaptive immune cells known to invade the stroma of tumor cells (Emmrich et al., 1998). CD3^+^ T lymphocytes mainly consist of CD4^+^ helper T cells, CD8^+^ cytotoxic T cells, and the regulatory T cells (Tregs). Interestingly, increased migration of Tregs in tumors and blood circulation is associated with a decrease in tumor progression (Hiraoka et al., 2006). The infiltration of Tregs in pancreatic tumors is mediated by binding of tumor-secreted chemokines to receptors on the Treg cell surfaces (Tan et al., 2009). In addition to Tregs, T helper cells play key roles in tumor cell growth or inhibition. While the response of type 2 T helper cells (Th2) is critical for imparting tolerance to tumors, the immune response of CD4^+^ T-cell-mediated type 1 T helper (Th1) is known to promote the death of pancreatic cancer cells (Tassi et al., 2008). Notably, in pancreatic cancer patients, an increased level of tumor-infiltrating CD4^+^ T cells is associated with improved survival (Ino et al., 2013). However, pancreatic cancer cells inhibit the proliferation and migration of CD4^+^ T cells (Fogar et al., 2011). Similarly, targeting the plasticity in Th1/2 subtype switch has shown promise as a candidate for cancer immunotherapy (Tassi et al., 2009). On the other hand, CD8^+^T cells comprise the most abundant population of T cells in pancreatic cancer, and a reduction in this T cell population is observed during the progression of pancreatic cancer (Ene-Obong et al., 2013; Shibuya et al., 2014). Specifically, pancreatic cancer cells inhibit the cytotoxic action of T cells by multiple mechanisms including inhibition of perforin and granzyme secretion, lack of expression of major histocompatibility complex (MHC) class I molecules, and expression of the programmed death-ligand 1 (PD-L1) by tumor cells that bind to the programmed cell death-1 (PD-1) to suppress the function of CD8^+^T cells (Dong et al., 2002; Ryschich et al., 2005; Thomas and Massague, 2005). Critical for the development of T cells is the cytotoxic T-lymphocyte-associated protein 4 (CTLA-4) receptor expressed on CD4^+^T and CD8^+^ T cells. Activation of the CTLA-4 receptor is required for suppression of the immune response by Treg cells, resulting in a decrease in cytotoxic T cells. Blockage of CTLA-4 mounts an effective immune response mediated by an increased cytotoxic T cell population and a decreased Treg population to suppress tumor progression (Johansson et al., 2016). Notably, the PI3K pathway is critical for the development, proliferation, and activation of T cells. For example, disruption of the PI3Kδ gene by a D910A-inactivating point mutation in mice impaired T and B cell receptor signaling, leading to a suppressed immune response (Okkenhaug et al., 2002). Furthermore, PI3K inhibitor treatment in a KPC mouse model of PDAC reduced disease pathology and metastasis, and prolonged survival (Figure 2). This immunoprotective effect in pancreatic lesions correlated with a decrease in Treg cells concomitant with an increase in CD44^high^CD8^+^ T lymphocyte population (Ali et al., 2014). PI3K-mTOR pathway has also been implicated in regulating the release of granzyme B by Treg cells. Deletion of PI3Kδ affected the secretory perforin-granzyme pathway leading to degranulation of cytotoxic T lymphocytes to impact tumor surveillance (Figure 2; Putz et al., 2012). Thus, targeting pathways of the immune response, with a particular focus on T cells, holds promise as a potential treatment strategy.

#### B cells

B cells are known to infiltrate tumor cells during PDAC progression and support the growth of cancer cells by suppressing CD8^+^ T cells and tumor-associated macrophages (TAMs). Likewise, inhibition or genetic deletion of B cells leads to the activation of CD8^+^ T cells that further inhibit tumor cell growth (Roghanian et al., 2016). A study conducted in a mouse model of PDAC showed that the stroma in PanIN lesions secretes the chemokine ligand 13 (CXCL13), a chemokine that attracts B cells to the tumor periphery. These recruited B cells promote the proliferation of transformed epithelial cells via interleukin-35 (IL-35)-mediated paracrine signaling (Pylayeva-Gupta et al., 2016). Further, HIF-1α deletion in the KC mouse model resulted in enhanced secretion of B cell chemoattractants that increased the population of the B cell subclass B1b in early pancreatic neoplasia. Notably, depletion of B cells has been shown to result in reduced progression of PanINs and tumorigenesis in the mice (Lee et al., 2016). In a study on Bruton tyrosine kinase (BTK) expressed in B cells and macrophages, this enzyme was shown to play a critical role in suppressing PDAC growth. Specifically, the B cell-macrophage crosstalk was demonstrated to reprogram TAMs to Th2 type via the activation of BTK in a PI3Kγ-dependent manner. Inhibition of either BTK or PI3Kγ was found to reprogram macrophages to Th1 type leading to activation of T cell-mediated immunity to control pancreatic cancer growth (Figure 2; Gunderson et al., 2016). Thus, the interconnected signaling pathways of B cell and TAMS are critical for regulating tumor growth.

### PI3K signaling in endothelial cells

Endothelial cells present in the tumor microenvironment are critical for regulating angiogenesis and maintaining vasculature inside tumors. Remodeling of the local tumor vasculature is critical for the exchange of nutrients and signaling molecules between the hypoxic core and the peripheral regions (Folkman, 2007). Pancreatic tumor cells secrete high levels of VEGF that in turn bind to VEGF receptors present on the endothelial cells (Yamazaki et al., 2008). This interaction activates PI3K signaling crucial for proliferation, survival, and maturation of endothelial cells (Figure 2; Luo et al., 2001). Apart from VEGF receptor, endothelial cells secrete various other receptors like TIE, PDGFR, FGR, and ERBB receptor tyrosine kinases, whose activation triggers PI3K signaling (Hofer and Schweighofer, 2007). Hence, PI3K signaling acts as a key regulator of angiogenesis and lymphatic vessel formation (Hamada et al., 2005).

## Targeting the inositide signaling pathway using chemical inhibitors

Constitutively activated mutant K-Ras signals via the MAPK, PI3K-Akt, NF-κB, WNT–β-catenin, Notch, and SMAD pathways. The existence of complex cross-signaling and feedback loops between these pathways remains one of the major factors in determining the development of resistance to therapeutic drug regimens. In addition to K-Ras-driven PI3K activation, aberrant expression of the PTEN protein results in constitutive activation of the PI3K and Akt signaling pathways in pancreatic cancer. In light of such dysregulation of the PI3K signaling cascade, intense research has been directed toward the development of inhibitors that target this critical node. Various classes of inhibitors have been developed that specifically target PI3K, Akt, and mTOR signaling pathways (Figure 1). Interestingly, many of these inhibitors are under clinical trials, thereby paving a new path for improved therapeutic approaches pertinent to the treatment of PDAC.

### Targeting PI3K

Multiple generations of PI3K inhibitors have been developed, many of which are under clinical trials (Table 2). Historically, the poor pharmacokinetic properties of various Pan PI3K inhibitors, such as Wortmannin and LY294002, has led to the evolution of next-generation PI3K inhibitors that include small molecule drugs, such as BKM120, GDC0941, GSK2126458, and RNA interfering (RNAi) agent, ATU027 (Khan et al., 2013). Despite the success of many of these drugs, one of the major challenges that contribute to the suboptimal response to monotherapies is the development of drug resistance. Multiple mechanisms underlying such resistance phenotypes have been reported which encompass activation of alternative signaling pathways, mutations in the secondary target, and amplification of downstream signaling moieties within the same pathway (Zahreddine and Borden, 2013). Under such scenarios, the identification of new targeted combination therapies is indispensable for developing a superior response to therapies to treat pancreatic cancer.

**Table 2:** Current ongoing clinical trials targeting the phosphoinositide signaling cascade for the treatment of pancreatic cancer.

**Target molecule**	**Drug**	**Clinicaltrials.gov identifier**	**Study phase**	**Intervention**
PI3K	Metformin	NCT01210911	Phase II	Metformin+Gemcitabine+Erlotinib
PI3K	BKM120	NCT01155453	Phase I	BKM120+ GSK1120212
PI3K	BKM120	NCT01363232	Phase I	BKM120 + MEK162
PI3K	BKM120	NCT01571024	Phase I	BKM120 + mFOLFOX6
Akt	MK2206	NCT01783171	Phase I	MK-2206+ Dinaciclib
Akt	MK2206	NCT01658943	Phase II	MK2206+ Fluorouracil+ Oxaliplatin+ Selumetinib
Akt	RX-0201	NCT01028495	Phase II	RX-0201+Gemcitabine
mTOR	Everolimus	NCT01077986	Phase I, II	Capecitabine + Cetuximab + Everolimus
mTOR	Temsirolimus	NCT00075647	Phase II	Temsirolimus
mTOR	Everolimus	NCT02294006		Everolimus+ Octreotide LAR+ Metformin
PI3K+mTOR	BEZ235	NCT01337765	Phase I	BEZ235 + MEK162
CDK4/6	Palbociclib	NCT03065062	Phase I	Palbociclib+Gedatolisib
EGFR	Nimotuzumab	NCT00561990, NCT02395016	Phase II/III	Gemcitabine ± nimotuzumab

*Data taken from https://clinicaltrials.gov/*.

While LY294002 monotherapy has low efficacy against tumors, the desired efficacy of this drug may be achieved by combination with the NSAID sulindac or gemcitabine, which improved the growth inhibitory effects of LY294002 by reducing the apoptotic threshold in PDAC cells (Yip-Schneider et al., 2003). A recent study has demonstrated that the suppression of the PI3K/Akt /mTOR pathway results in a compensatory activation of the MAPK/MEK pathway. The authors further established a reduction in the viability of pancreatic cancer cell lines by application of a dual-acting agent using the PI3K inhibitor, ZSTK474, and the Raf/MEK inhibitor, RO5126766 (Van Dort et al., 2015). Interestingly, MEK inhibition by AZD6244 alone remained cytostatic until it was combined with the PI3K-inhibitor BKM120 or erlotinib, which delayed tumor formation and improved overall survival compared with single-agent therapy (Alagesan et al., 2015; NCT01222689). A phase I study of BKM120 in combination with mFOLFOX6 in patients with metastatic pancreatic cancer is ongoing (NCT01571024). Gemcitabine has been the standard first-line treatment for patients with advanced or metastatic pancreatic cancer (Von Hoff et al., 2013). The therapeutic potential of gemcitabine can be improved by evodiamine, which negatively regulates the NF-κB signaling by targeting the PI3K/Akt pathway (Wei et al., 2012). Thus, targeting PI3K pathways by a combination of agents may be crucial for achieving the desired efficacy against tumors.

### Targeting Akt

Akt signaling is pertinent to the activation of anti-apoptotic pathways that support cancer cell survival. Akt inhibitors have been classified as ATP-competitive, allosteric, and Akt-protein-substrate-binding-site inhibitors. However, due to the extensive similarity of the ATP binding pocket of the Akt kinases to the AGC family of kinases, it has been particularly difficult to develop ATP-competitive inhibitors that target Akt kinases (Scheid and Woodgett, 2001). A recent report by Yap et al. assessed the effect of MK-2206, an oral allosteric inhibitor of all Akt isoforms, for anti-tumor activity in preclinical models. Their study showed a reduction in tumor size and a decrease in cancer antigen levels in a PDAC patient treated with 60 mg of the drug on alternate days (Yap et al., 2011). The drug has further been demonstrated to function along with dinaciclib, a cyclin-dependent kinase inhibitor, to abolish tumor growth in pancreatic cancer models (Hu et al., 2015). In addition to small molecule inhibitors, the emergence of antisense nucleotides that target key signaling molecules is promising. RX-0201, an Akt antisense oligonucleotide, has been tested in combination with gemcitabine in metastatic pancreatic cancer and is in phase II trials (NCT01028495).

### Targeting mTOR

The mTOR kinase, one of the key downstream effectors of K-Ras and the PI3K pathway, coordinates distal metabolic features, such as the presence of growth factors, to cell survival, growth, and proliferation by regulating various transcriptional and translational regulatory programs (Utomo et al., 2014). Rapamycin (sirolimus), one of the most long-standing inhibitors of mTOR, has shown broad anticancer activity (Douros and Suffness, 1981; Garber, 2001; Utomo et al., 2014). In the recent years, multiple rapamycin analogs have been developed, including everolimus, temsirolimus, and deforolimus (Hudes et al., 2007; Motzer et al., 2008). The oral mTOR-inhibitor everolimus is in phase 2 trials to treat advanced pancreatic neuroendocrine tumors and has shown antitumor activity by prolonging progression-free survival in patients (Yao et al., 2011). Meanwhile, researchers have developed inhibitors to target EGFR, which is frequently overexpressed in PDAC patients. The EGFR-inhibitor erlotinib tested in combination with rapamycin significantly disrupted the PI3K-Akt-mTOR signaling cascade producing a synergistic effect on cell growth inhibition (Buck et al., 2006).

Taken together, previous *in vitro* screens reveal that PDAC cell lines are relatively resistant to single-agent therapies and thus, targeting multiple nodes of the PI3K-Akt-mTOR signaling cascade using combination drug therapies might reinforce the response to individual drug therapies. The PI3K pathway status may serve as genetic determinants of therapeutic response in clinical trials.

## Conclusion and future perspective

Aberrant signaling pathways are an important hallmark of cancer and essential for development and progression of tumors. Signaling networks are activated in cancer cells upon sensing the molecular cues from outside the cells to regulate cellular functions (Jones et al., 2008). Cancer cells and surrounding cells present in the tumor microenvironment continuously interact by modifying the signaling cues during the different stages of cancer evolution. These dynamic cues are perceived by cellular signaling networks to impart adaptation to the tumor cells during the disease progression (Liu et al., 2017). K-Ras and PI3K are key regulated nodes in the complex, inter-connected signaling networks altered during pancreatic cancer (Jones et al., 2008). K-Ras is the key driver mutation in pancreatic adenocarcinomas and is required for initiation, progression, and maintenance of the disease (Bryant et al., 2014; Cancer Genome Atlas Research Network Electronic Address and Cancer Genome Atlas Research, 2017). PI3K mutations, although rarely found in pancreatic cancer compared to other forms of cancer, can contribute to tumorigenesis in a small subset of pancreatic cancer patients (Schonleben et al., 2006; Payne et al., 2015). Targeting PI3K in this small cohort of patients can be of great significance in this lethal malignancy. Because PI3K can be regulated by a myriad of growth factor receptors and pathways including K-Ras, it dispenses its effector functions by differentially regulating a multitude of downstream signaling cascades. PI3K signaling is directly activated by mutations in the K-Ras gene in pancreatic cancer (Eser et al., 2013). Despite the lack of significant genomic alterations in the PI3K pathway, it is still considered a key node in the signaling network considering that it is interconnected with other signaling pathways at multiple levels. Co-targeting the PI3K pathway with multiple other signaling pathways and known drugs has seen limited success, but it remains a viable strategy given that multiple PI3K inhibitors are in human clinical trials (Bowles and Jimeno, 2011). It would be beneficial for future research to be targeted to understand PI3K-inhibitor unresponsiveness and resistance, and eventually design novel combinatorial drug strategies using resistance-pathway inhibitors and other targets.

A second critical aspect of pancreatic cancer research is the interaction of tumor cells with the surrounding cells present in the tumor microenvironment. Recent studies not only highlight the importance of PI3K signaling in development and maintenance of immune and stromal cell functions but also in modifying signaling cues between tumor and accessory cells during the progression of cancer (Koyasu, 2003; Polo et al., 2015; Gunderson et al., 2016). It is now well-established that both tumor cells and the tumor microenvironment continuously interact to modify the outcome during tumor development and changing the properties of one component modifies the other (Whatcott et al., 2015). While the genome of a cancer cell continues to evolve, thus making it hard to target, the stromal or immune cells, on the other hand, harbor a robust and stable genome. Therefore, targeting signaling pathways in these accessory cells can yield notable results in cancer prevention and disease control. PI3K has been examined in the tumor microenvironment to a certain extent; a comprehensive understanding of all the signaling pathways that include PI3K will need to be elucidated in the future. Hence, understanding the oncogenic mechanisms during tumor-stromal and tumor-immune crosstalk will be of paramount importance to dissect the pathways critical for pancreatic cancer pathogenesis. This will aid in designing multi-drug, multi-target combination therapies and will prove critical in systematically screening patient biopsies for both driver mutations and other downstream-activated signaling pathways, like PI3K, in order to design personalized therapies.

## Author contributions

DM and KA: made figures; DM, KA, and PS: wrote the manuscript.

### Conflict of interest statement

The authors declare that the research was conducted in the absence of any commercial or financial relationships that could be construed as a potential conflict of interest.

## Abbreviations

4E-BP: eIF4E-binding proteins
ACLY: ATP citrate lyase
ADM: acinar-to-ductal metaplasia
BTK: Bruton tyrosine kinase
CAT: Catalytic domain
CTLA: Cytotoxic T-lymphocyte-associated protein
CXCL13: Chemokine ligand 13
eIF4E: eukaryotic translation initiation factor 4E
ERK: Extracellular signal-regulated kinases
EXT: Extension domain
FoXO: Forkhead transcription factors
GCPR: G-protein-coupled receptors
GSK3β): glycogen synthase kinase 3 beta
GTPases: Guanosine-5′-triphosphatases
HIF-1α: Hypoxia-inducible factor 1- α
HK: Hexokinase
HM: Hydrophobic motif
HNRNPA2B1: Heterogeneous nuclear ribonucleoprotein A2/B1
IFNγ: Interferon gamma
MAPK: Mitogen-activated protein kinase
MHC: Major histocompatibility complex
mTOR: Mechanistic target of Rapamycin
MUC: Mucin
NF-κB: Nuclear factor kB
NK: Natural killer
P70S6K: ribosomal protein S6 kinase beta-1
PanINs: Pancreatic intraepithelial neoplastic lesions
PD-1: Programmed cell death-1
PD-L1: Programmed death-ligand 1
PDAC: Pancreatic ductal adenocarcinoma
PDK1: Phosphoinositide-dependent kinase 1
PH: Pleckstrin homology
PI3K: Phosphoinositide-3-kinase
PKB: Protein kinase B
PtdIns: Phosphatidylinositols
PTEN: Phosphatase and tensin homolog deleted on chromosome 10
Ras: Rat sarcoma virus
ROS: Reactive oxygen species
RTKs: Receptor tyrosine kinases
SH2: Src Homology 2
TAM: Tumor-associated macrophages
TGF-β: Transforming growth factor beta
Th: T helper
Treg: Regulatory T cells
VEGF: Vascular endothelial growth factor.
